# Identifying subgroups of persons with multimorbidity based on their needs for care and support

**DOI:** 10.1186/s12875-019-1069-6

**Published:** 2019-12-27

**Authors:** Mieke Rijken, Iris van der Heide

**Affiliations:** 1 Nivel (Netherlands Institute for Health Services Research), PO Box 1568, 3500 BN Utrecht, The Netherlands; 2 Department of Health and Social Management, University of Eastern Finland, P.O. Box 1627, FI-70211 Kuopio, Finland

**Keywords:** Multimorbidity, Needs, Patient segmentation, Integrated care, Quality of life

## Abstract

**Background:**

There is broad consensus that countries need to develop and implement person-centred integrated care to better meet the needs of their growing populations with multimorbidity. To develop appropriate care, it is essential to know the needs for care and support among these populations. For this purpose, we examined whether subgroups of people with multimorbidity could be distinguished based on their needs, and profiled these subgroups according to medical complexity and the availability of personal resources.

**Methods:**

Persons diagnosed with two or more somatic chronic diseases (*N* = 613) were selected from 38 general practices throughout the Netherlands. We conducted a cluster analysis of their scores on the RAND-36 questionnaire of health-related quality of life (QoL), to gain insight in their needs for care and support. Differences in demographics, medical characteristics and personal resources between the identified clusters were tested using analysis of variance and chi-square tests.

**Results:**

The cluster analysis revealed three subgroups: 1. a group with a relatively good QoL (48% of the sample), 2. a group with a poor physical QoL (28%), and 3. a group with a poor QoL in all domains assessed by the RAND-36 (24%). The group with a relatively good QoL had more favourable medical characteristics than the other groups, i.e., less chronic diseases, shorter illness duration, more stable course of illness, better controllable conditions, less polypharmacy. The group with a poor QoL in all domains could rely on less personal resources (education, income, social support, health literacy, self-management capabilities) than the other groups.

**Conclusions:**

Different subgroups of people with multimorbidity can be distinguished based on their needs for care and support. These needs are not only determined by demographic and medical characteristics, but also by the personal resources people have available to manage their health and care. Patient profiles combining medical complexity and personal resources could guide the development of integrated care for specific target groups of persons with multimorbidity.

## Background

An increasing number of people worldwide suffer from multimorbidity, i.e. the co-occurrence of two or more chronic diseases within a person [1]. Especially among older people the prevalence of multimorbidity is high [2], and although estimates vary, among primary care patients older than 65 multimorbidity now seems the rule rather than the exception [3].

Multimorbidity asks for new models of organizing care, as many people with multimorbidity need care from multiple care providers from different disciplines [4, 5], who need to work as a solid team overcoming professional and organizational boundaries [6]. Some people with multimorbidity may have highly complex medical needs, which require close collaborations between different medical disciplines in primary care and hospitals. Others do not have very complex medical problems, but experience functional disability and participation problems in daily life. In such cases, social care may be necessary, in addition to (primary) healthcare. Discipline- and sector-encompassing needs cannot be adequately met without good coordination and collaboration at the clinical level, supported by organizational structures, financing methods and legislation that facilitate care integration [7].

Until now, integrated care has predominantly been designed and implemented to manage well defined diseases, for instance by chronic disease management programmes or oncological care pathways. However, multimorbidity is not a single, clearly demarcated condition and, as such, disease-centred care programmes are not suitable. This implies that integrated care for people with multimorbidity should be person-centred, taking their individual needs, goals and preferences as a starting point of their individual care plans [8, 9]. The development of individual care plans could be guided by flexible (non-directive) integrated care arrangements made by all stakeholders at the local level.

**Fig. 1:**
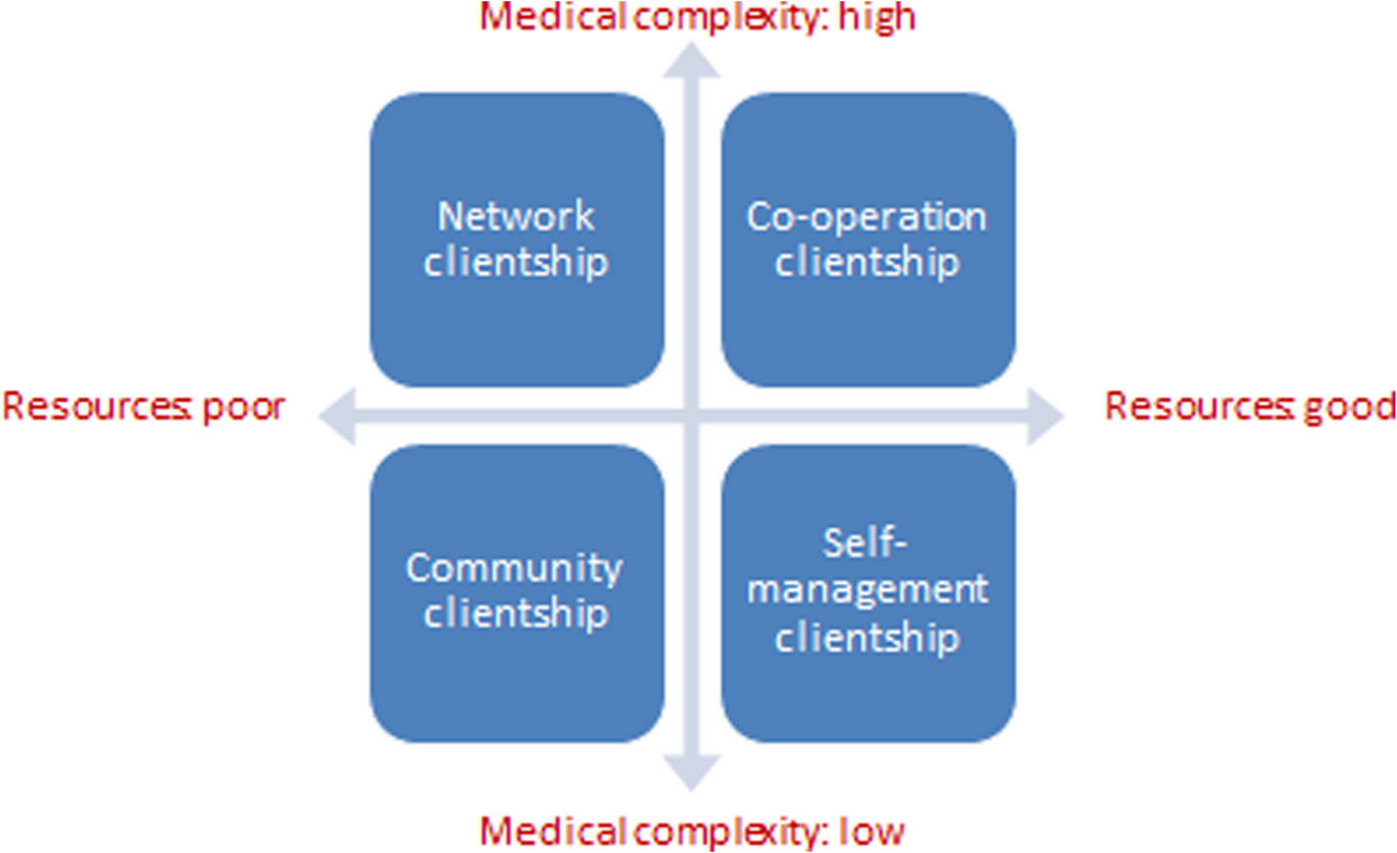
Clientship model (Source: Koivuniemi et al., 2014; Hujala et al., 2017)

To guide the development of such integrated care arrangements, Koivuniemi and colleagues [10] present a simple framework distinguishing chronic patients with different needs for care and support, based on two dimensions: 1) the complexity of the medical condition(s), related treatments and services needed, and 2) the resources the individual has to manage his health and care. Combining these dimensions results in four patient or ‘clientship’ profiles (Fig. 1): 1. Self-management clientship: medical condition not complex and sufficient resources such as knowledge and skills or family support to manage one’s health and care; 2. Community clientship: medical condition not complex but limited resources; 3. Co-operation clientship: medical condition complex but sufficient resources to co-operate with care professionals and contribute to care, for instance, by self-monitoring or self-treatment; and 4. Network clientship: medical condition complex and limited resources. According to the Finnish developers of this framework, the four profiles indicate which form of care integration is most appropriate: horizontal integration (Community clientship), vertical integration (Co-operation clientship) or both, i.e., comprehensive integration, (Network clientship). The patient profiles could also be used to target and allocate services.

Until now, no empirical evidence exists of the occurrence of the four patient profiles among multimorbid populations. Moreover, hardly any research has been conducted that provides insight into “strata” of people with multimorbidity with various needs for care and support [11], as most studies focus on specific subgroups, such as frail older people or people with a specific combination of diseases. With this study we aim to fill this gap, by 1) exploring which subgroups of people with multimorbidity could be distinguished based on their needs for care and support, and, 2) describing the demographic and medical characteristics and personal resources of the people belonging to these subgroups. In this way, we provide insight in whether there is empirical support for the patient profiles distinguished by the clientship framework. This could help to decide about the usefulness of the framework as a basis for developing integrated care for various multimorbid populations at a local level.

## Methods

### Study design, sample and data collection

We conducted an observational study, analyzing survey data from people with multimorbidity who participated in a nationwide panel-study in the Netherlands [12, 13]. Participants with chronic diseases are recruited each year from (random samples of) general practices in the Netherlands according to the following criteria: a diagnosis of somatic chronic disease(s), aged ≥15 years, not being institutionalized, life expectancy > 6 months (according to the general practitioner), being mentally able to participate, and having sufficient mastery of the Dutch language. Participants fill in questionnaires in April and October, for a maximum of four years.

In April 2015, 1194 persons with chronic disease(s) participated in a survey (80% of 1497 invited panel members), of whom 613 had been diagnosed with two or more chronic diseases. These 613 persons, registered with 38 general practices, constitute our study sample. Additional data provided by these respondents in surveys of October 2014 (*N* = 429) and October 2015 (*N* = 439) were also analysed. The main reason for the lower numbers of respondents on these two measurements is that annually a quarter of all panel members are replaced because of reaching the maximum participation term of four years. Therefore, not all panel members who responded in April 2015 already participated in the panel-study in October 2014, and not all of them were still participating in October 2015.

### Measures

#### Needs

In April 2015 respondents completed the Dutch validated version of the RAND-36 Short-form Health Status Survey [14, 15]. The RAND-36 assesses a person’s health-related quality of life, which could be used as an indicator of needs [16, 17]. The questionnaire consists of eight scales: general health, health changes, physical functioning, role limitations because of a physical problem, bodily pain, vitality, mental health, social functioning, and role limitations because of an emotional problem. Scale scores range from 0 (poor) to 100 (excellent). Cronbach’s alphas of the scales reported from a Dutch general population sample varied between ·71 and ·92 [15]. Cronbach’s alphas in this study ranged from ·79 to ·93.

#### Medical characteristics

At inclusion in the panel-study, general practitioners provided the following data (with permission of the participants):

- *Chronic disease types*: retrieved from participants’ health records (registered as ICPC codes [18]) and categorised as: cardiovascular disease, lung disease, diabetes, cancer, musculoskeletal disease, digestive disease, neurological disease or other disease.
- *Number of chronic diseases*: categorized as two, three and four or more.
- *Illness duration*: computed from the month and year of diagnosis of participants’ first chronic disease.
- *Evaluation of health condition*: GPs assessed the overall health condition of the participants on four dimensions; to what extent it was: 1) life-threatening, 2) progressively deteriorating, 3) showing an intermittent course, and 4) medically controllable. Scores on these items could range between 1 (to a less extent) and 3 (to a large extent).

In addition to the data provided by the general practitioners, participants themselves reported whether they used five or more different types of medicines in October 2015. This was included as a measure of *polypharmacy* (yes/no).

#### Personal resources

Information about participants’ resources was based on self-report. Most measures were included in the survey of April 2015, apart from scales assessing loneliness and health literacy (included in the survey of October 2014) and self-management capabilities (included in the survey of October 2015).

- *Education level*: based on the highest level of completed education. We distinguished three categories: low (primary school or low/preparatory vocational training), intermediate (intermediate or advanced general education or intermediate vocational training), and high (high vocational education or university).
- *Social resources*: Participants reported whether they lived together with one or more other adults (1) or not (0). This variable is referred to as *living situation*. In addition, we used one item of the Loneliness scale [19]: “There are plenty of people I can lean on when I have problems”, with three response options (yes, more or less, no). We refer to this variable as *perceived available support*, dichotomized as ‘yes’ (1) versus ‘more or less’ and ‘no’ (0).
- *Financial resources*: Participants’ *financial situation* was assessed by the item: “How is your financial situation at the moment?” (answering options: 1: I have to make debts, 2: I need to use my savings, 3: I get by, 4: I save some money, and 5: I save a lot of money). In addition we included a measure of *social deprivation* [20] consisting of seven items, which refer to buying new clothes regularly, possessing a car, inviting friends or relatives for dinner, going out, going on a holiday every year, membership of a club (e.g. fitness club, music classes) and equipment for leisure time activities (e.g. sporting equipment or a new bicycle for the children). Each item is scored 0 or 1, where a score of 1 indicates that a person did not perform the activity or did not possess the item because of a lack of financial means. Sum scores are calculated, ranging from 0 to 7. A score of 3 or higher refers to being socially deprived [20].
- *Health literacy*: We used three screening questions, developed by Chew and colleagues [21] and validated in a Dutch sample [22]: 1) a question referring to how often a person needs help to read materials provided by a care professional or organization; 2) a question referring to how confident a person is that he fills out medical forms adequately; and 3) a question referring to how often a person experiences problems learning about his medical condition because of difficulty understanding written information. Each question has five response options, ranging from 1 to 5. Higher scores indicate more problems, thus lower health literacy.
- *Self-management capabilities*: Participants completed the Partners in Health 12-item scale (PIH) [23]. The 12 items refer to perceived knowledge and abilities as well as to actual behavioural self-management (e.g., self-monitoring of symptoms, shared decision-making). The Dutch version was found to consist of two subscales in a sample of persons with COPD [24], but as the dimensionality of the PIH has proven to be different in other studies and countries, we computed one total (average) score, which could range between 0 and 8. Higher scores indicate better self-management capabilities. Cronbach’s alpha was ·81.

### Statistical analysis

To distinguish subgroups of people with multimorbidity, we conducted K-means cluster analysis of the eight RAND-36 scale scores. K-means is a non-hierarchical cluster analysis, using an iterative procedure that randomly partitions observations into a pre-set number (*k*) of clusters. Observations are assigned to the nearest cluster based on their distance from the initial cluster means. After assigning all observations to a cluster, the cluster means are recomputed on the basis of its member cases, and the assignment of cases to the nearest cluster is repeated. This iterative process is repeated until no more cases move from one cluster to another. We performed a series of cluster analyses by setting *k* = 2, *k* = 3, *k* = 4, *k* = 5 and *k* = 6. The most meaningful number of clusters was determined by the criterion of Calinski and Harabasz: a higher score on this criterion indicates a better fit of the cluster structure [25]. Subsequently, ANOVA’s and posthoc analyses using the Scheffé test were applied to assess the statistical significance of differences between the identified clusters on the RAND-36 scales.

To test for differences between the clusters in the demographic and medical characteristics as well as the personal resources of the persons belonging to these clusters, we conducted ANOVA’s with post hoc Scheffé tests (in case of continuous variables) and chi-square tests (in case of categorical variables).

## Results

### Sample characteristics

The sample (*N* = 613) consisted of slightly more men (53·2%) than women (46·8%). The mean age was 68·3 years (SD 10·9). Among the total sample 61·3% had been diagnosed with two chronic diseases, 24·5% with three, and 14·2% with four or more chronic diseases. The mean illness duration was 15·1 years (SD 10·1).

The mean scores on the RAND-36 scales varied between 41·5 for health changes and 71·4 for mental health. The mean score on the health changes scale (< 50) indicates that on average participants perceived a deterioration of their health over the last year. The mean score on the mental health scale indicates a relatively good mental health.

### Subgroups of people with multimorbidity

**Fig. 2:**
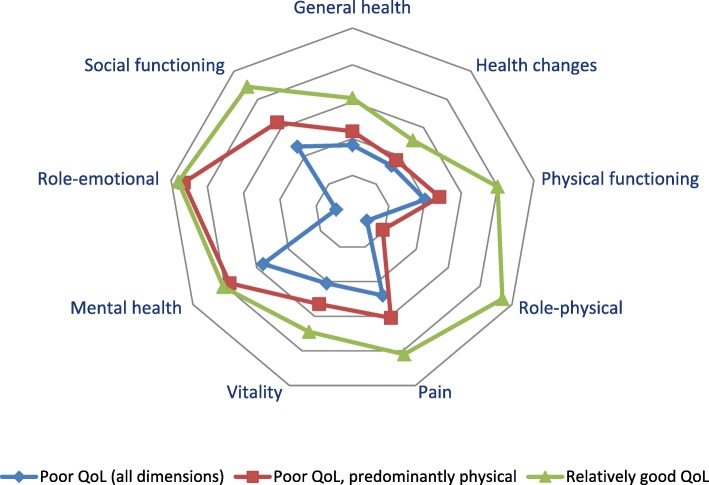
Visualisation of the three clusters of multimorbid persons; mean scores of the RAND-36 dimensions of cluster 1 (‘poor QoL (all dimensions’), cluster 2 (‘poor QoL, predominantly physical’) and cluster 3 (‘relatively good QoL’)

The cluster analysis resulted in three subgroups (Table 1): a group of 131 persons (24·2%) with relatively low scores on all eight scales (cluster 1, labelled ‘poor QoL, all dimensions’), a group of 149 persons (27·5%) who have relatively low scores on the scales referring to physical QoL, but much higher scores on the scales referring to their mental health and emotional wellbeing (cluster 2, labelled ‘poor QoL, predominantly physical’) and a larger group of 262 persons (48·3%) who have relatively high scores on all eight scales (cluster 3, labelled ‘relatively good QoL’). Post-hoc analyses revealed that people belonging to the first cluster had significantly lower scores on all RAND-36 scales, except on the ‘health changes’ scale, than people belonging to the second cluster (p < ·001). And people assigned to cluster 2 scored significantly lower on all scales, except on the scale ‘role limitations because of emotional problems’, than people belonging to the third cluster 3 (*p* < ·001). Fig. 2 visualizes the three subgroups distinguished by their RAND-36 scores.

**Table 1:** Description of subgroups of people with multimorbidity (clusters), according to their mean scores on the RAND-36 scales (*N* = 542)

	Cluster 1 (*n* = 131)	Cluster 2 (*n* = 149)	Cluster 3 (*n* = 262)	ANOVA	*P*-value
	*M* (SD)	*M* (SD)	*M* (SD)	*F* (2, 539)	
General health	36.5 (17.1)	44.0 (17.0)	62.2 (16.1)	123.37	<.001
Physical functioning	39.7 (22.9)	48.1 (23.5)	80.4 (17.9)	211.04	<.001
Mental health	56.2 (18.9)	76.8 (13.0)	81.2 (12.5)	135.44	<.001
Pain	48.2 (25.0)	61.0 (20.5)	82.0 (16.7)	137.33	<.001
Emotional role functioning	8.7 (15.2)	92.6 (15.4)	95.9 (14.9)	1611.17	<.001
Physical role functioning	9.0 (19.9)	19.3 (22.3)	94.1 (13.3)	1363.45	<.001
Social functioning	46.6 (22.0)	63.7 (19.1)	89.0 (13.4)	279.14	<.001
Health changes	33.4 (19.0)	36.6 (21.3)	51.0 (14.6)	55.15	<.001
Vitality	41.1 (17.3)	53.4 (15.0)	69.4 (14.3)	160.16	<.001

### Demographic characteristics of the subgroups

Table 2 shows that the three subgroups differed with respect to their gender distribution. Cluster 1 (‘poor QoL, all dimensions’) consisted of significantly more women than the other two clusters. Surprisingly, the three subgroups did not differ in age. The proportion of participants with non-western roots was low and did not differ between the subgroups.

**Table 2:**
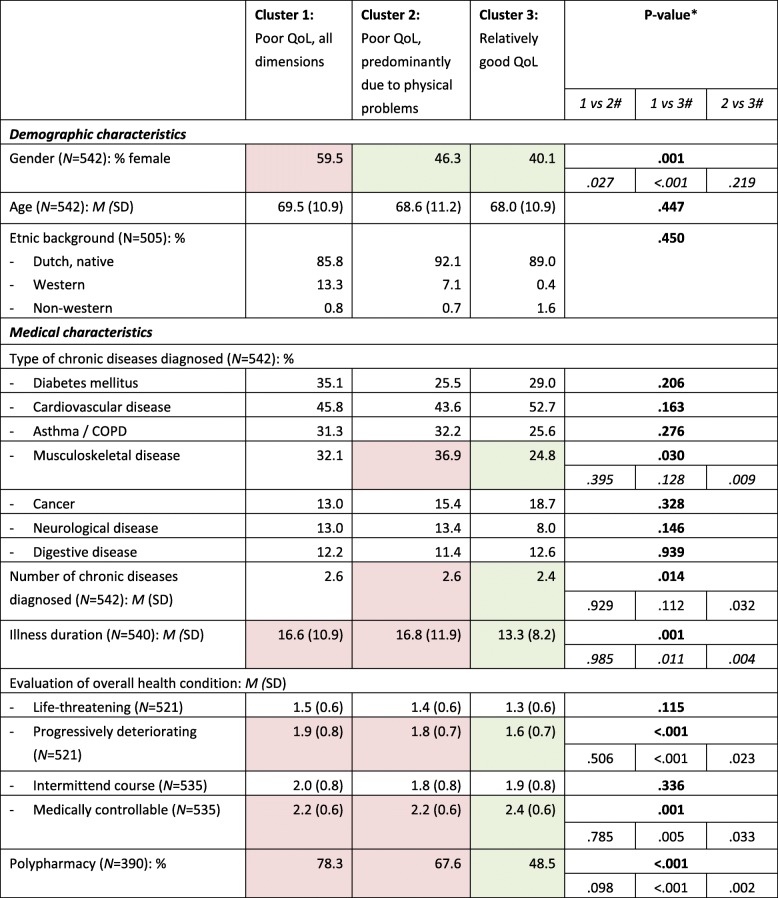
Demographic and medical characteristics of subgroups of people with multimorbidity

* Results of Chi^2^-test for categorical variable or F-test for continuous variable across all three subgroups

# Results of post-hoc Chi^2^-test or post-hoc Scheffé test between two subgroups, in case of significant Chi^2^- or F-test across all three subgroups

Mean or distribution in red cells differ from mean or distribution in green cells.

### Medical characteristics of the subgroups

Apart from the presence of musculoskeletal diseases, no differences between the three subgroups existed in the distribution of chronic disease types (Table 2). Musculoskeletal diseases were more prevalent in cluster 2 (‘poor QoL, predominantly physical problems’) than in cluster 3 (‘relatively good QoL’).

Other medical characteristics seemed to be more distinctive than disease type. People assigned to cluster 2 (‘poor QoL, predominantly physical problems’) had more chronic diseases than people in the third cluster (‘relatively good QoL’). On all other medical characteristics persons assigned to the cluster with a relatively good QoL significantly differed from persons assigned to both other clusters: they had a shorter illness duration, used five or more different medicines less often and their health condition was less often deteriorating or uncontrollable.

#### Resources of the subgroups

Table 3 shows that differences in available resources are more often seen between persons assigned to cluster 1 (‘poor QoL, all dimensions’) and persons belonging to cluster 2 or 3. In cluster 1 there are more people with a low education level, a less favourable financial situation, more people who report difficulty understanding written health information and more people with less self-management capabilities. People in cluster 1 also differ from people in cluster 3 in their perception of available support and other aspects of health literacy. In the first group less persons perceive they have support available when needed, and more persons experience problems reading medical information and filling in medical forms.

**Table 3:**
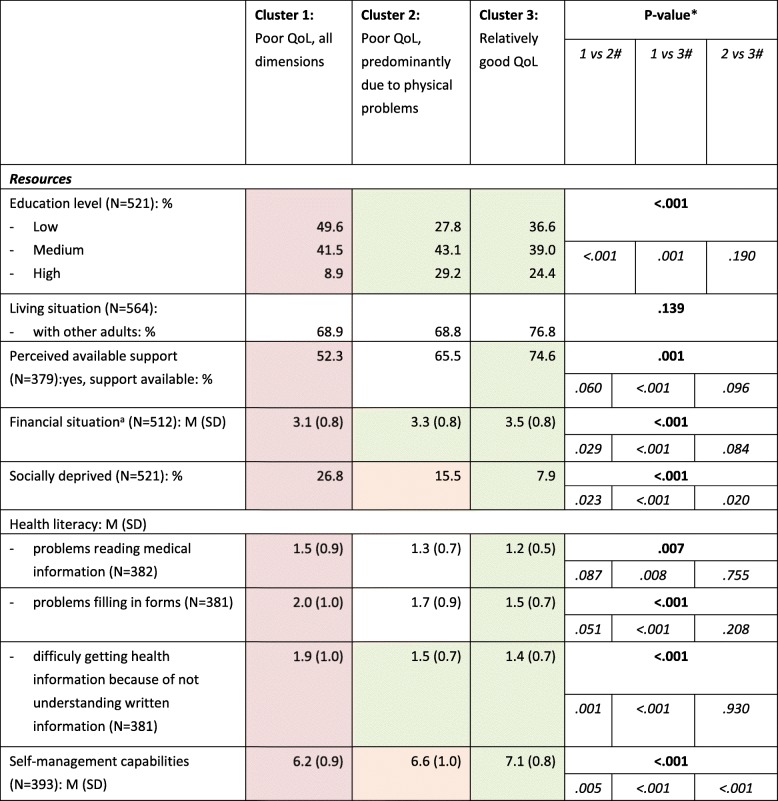
Personal resources of subgroups of people with multimorbidity (clusters)

* Results of Chi^2^-test for categorical variable or F-test for continuous variable across all three subgroups

# Results of post-hoc Chi^2^-test or post-hoc Scheffé test between two subgroups, in case of significant Chi^2^- or F-test across all three subgroups

^a^ Higher scores indicate more financial security

Mean or distribution in red cells differ from mean or distribution in green cells; mean or distribution in orange cells differ from mean or distribution in red and green cells.

## Discussion

### Discussion of the main findings

**Fig. 3:**
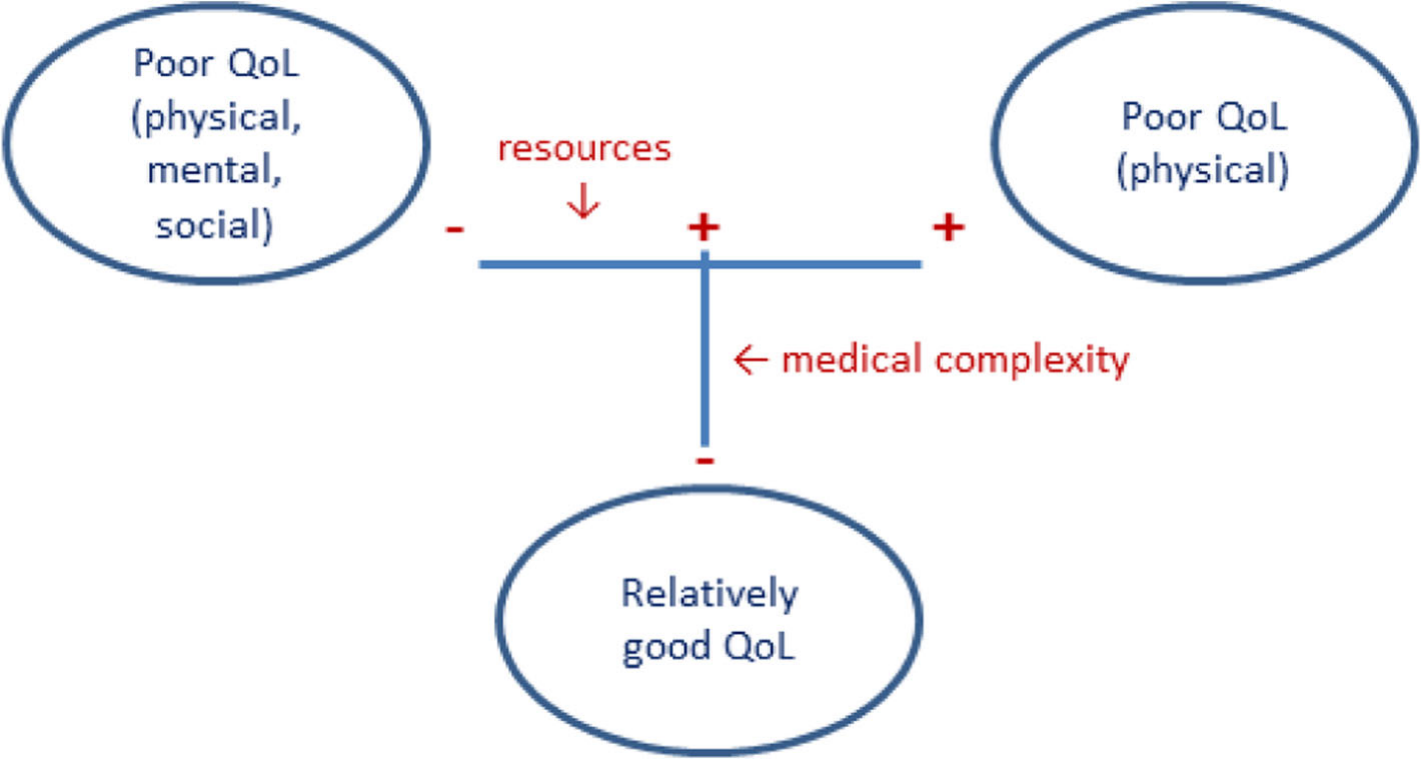
Illustration of the positioning of the three groups of people with multimorbidity according to the medical complexity of their health condition and their resources

Among a Dutch sample of people with multimorbidity, we found three subgroups with different needs for care and support. The largest group (48%) reported a relatively good QoL, with similar or even higher scores on the RAND-36 scales, except the general health scale, than a Dutch general population sample [15]. This group had more favourable medical characteristics than the two other subgroups of people with multimorbidity, and also more personal resources than the group with a poor QoL (all RAND-36 scales). The second group in size (28%) consists of people who report a good mental health and few role limitations because of emotional problems, but many role limitations due to physical problems as well as pain and functional disability, a lack of energy, a poor general health and negative health changes. This group, labelled as ‘poor QoL, predominantly physical problems’, differs from the group with a relatively good QoL in medical characteristics (less favourable) but not in available resources. The third group, slightly smaller in size (24%), consists of people who have low scores on all RAND-36 scales. Besides their physical problems, these people experience many role limitations due to both physical and emotional problems and they also report problems with social functioning. People assigned to this third group resemble the people in the second group regarding their medical characteristics, but differ from the latter regarding their resources: they are lower educated, have less financial means, more problems understanding health information and less self-management capabilities. In summary, medical characteristics distinguish the first group from the second and third, whereas the availability of resources distinguishes the third group from the first and second (see Fig. 3).

### Limitations and considerations

We used participants’ RAND-36 scores as indicators of their needs, which has been done in other studies as well [16, 17]. Needs assessed in this way may not fully correspond with the concept of care and support needs as meant by Koivuniemi and colleagues in the Clientship model [10]. With some reservations we therefore suggest that our results partly support the clientship profiles distinguished by the model based on both the medical complexity of a person’s health condition and his or her personal resources. However, as our sample solely consisted of people with multimorbidity, it could be argued that none of the participants had a health condition that was not medically complex, and so the pure self-management and community clientship profiles of the framework may not fully apply to persons with multimorbidity. Nevertheless, care models that put more or less emphasis on self-management and involvement of community services are relevant for subpopulations of people with multimorbidity as well.

In addition, it should be noticed that mental health conditions were not taken into account as chronic conditions in the panel-study. This means that persons were only included if they had been diagnosed with at least two somatic chronic diseases. This implies that people with one somatic chronic disease in combination with mental health disorders or social problems were not included. From general practitioners we know that they see many patients who do not meet our strict criteria of multimorbidity but who have mental health or social problems in addition to a somatic chronic disease, for which they visit the practice. It is likely that many of these patients lack sufficient resources to manage their health and care, and as such may benefit in particular from integrated health and social care.

We do not know whether the distribution according to the three clusters we found in our sample would also be found in other samples or populations of people with multimorbidity. In a study using the EQ-6D instead of the RAND-36, four clusters of multimorbid people were distinguished: a group with a relatively good QoL (52%), a group experiencing problems with mobility, performing daily activities and pain/discomfort (39%), a small group (4%) with similar problems as the second cluster but also experiencing anxiety/depression and problems with self-care, and another small group (4%) with predominantly cognitive problems [26]. This distribution shows some similarities with the distribution found in this study, but it should be noted that both studies included participants from the same panel-study. However, finding other proportions does not necessarily point to low external validity, as prevalence rates of chronic diseases and disease patterns as well as available resources of populations vary across regions and countries. This is exactly why care providers at local level should assess the profiles of their multimorbid populations as a basis for developing integrated care.

Finally, we wish to point to multiple testing: Tables 2 and 3 contain the results of 26 Chi^2^- or F-tests to test for differences in demographics, medical characteristics and resources across the three clusters. Applying Bonferroni correction (α/k), would result in a significance level of .0019, indicating that *P*-values greater than or equal to .002 would no longer be considered significant. This would mean that the three clusters may not be different regarding the presence of musculoskeletal disease and the number of chronic diseases (Table 2) or their experience of problems reading medical information (Table 3).

### Implications for developing integrated care and clinical practice

Based on the profiles of their patients with multimorbidity, multidisciplinary groups of care professionals in collaboration with patient and family representatives could develop integrated care at a local level for different target groups. This has been done in the Finnish Pirkanmaa region, where the clientship profiles were used to develop a coherent set of integrated care services, including supportive tools for professionals and patients and assigning responsibility for care coordination. Recently, several frameworks [27, 28] have been published that could guide the development of people-centred integrated care for target populations with multimorbidity at the local level. In addition to developing people-centred integrated care programmes based on patient profiles, it is essential to adequately support the implementation of these programmes. This not only includes training of care professionals in exploring and assessing patients’ needs and resources in dialogue with patients or family members, but also redesigning care delivery processes to allow more flexibility for tailoring care to individual patients’ needs. Health systems need to be reformed to shape the right conditions for people-centred integrated care [29].

Independent of using the clientship framework, our results provide guidance for managing multimorbidity in clinical practice. This starts with a regular assessment of the care needs of patients with multimorbidity, not only based on medical data but also on patients’ information about their personal resources. Our study shows the value of assessing patients’ resources, in particular the availability of social support, health literacy and self-management capabilities. Financial barriers for health service use or access to community support may also be considered for inclusion in such assessments. Together with patients’ reports of their personal goals and values, assessments of their care needs could guide the development and follow-up of individual care plans, supported by people-centred care delivery systems.

## Conclusions

Different subgroups of people with multimorbidity can be distinguished based on their needs for care and support. These needs are not only determined by demographic and medical characteristics, but also by the personal resources people have available to manage their health and care. Patient profiles combining medical complexity and personal resources could guide the development of integrated care for specific target groups of persons with multimorbidity.

## Data Availability

The dataset analysed for the current study is available from the corresponding author on reasonable request.

## Abbreviations

COPD: Chronic Obstructive Pulmonary Disease
GP: General Practitioner
ICPC: International Classification of Primary Care
PIH: Partners in Health
QoL: Quality of Life
